# Modulation of pro-inflammatory activation of monocytes and dendritic cells by aza-bis-phosphonate dendrimer as an experimental therapeutic agent

**DOI:** 10.1186/ar4546

**Published:** 2014-04-18

**Authors:** Yannick Degboé, Séverine Fruchon, Michel Baron, Delphine Nigon, Cédric Olivier Turrin, Anne-Marie Caminade, Rémy Poupot, Alain Cantagrel, Jean-Luc Davignon

**Affiliations:** 1 Centre de Rhumatologie, CHU PURPAN, 1 Place Baylac, 31300 Toulouse, France; 2 INSERM U1043 - CNRS UMR 5282, CPTP, Université Paul Sabatier, Toulouse, France; 3 Laboratoire de Chimie de Coordination, CNRS, Toulouse, France

## Abstract

**Introduction:**

Our objective was to assess the capacity of dendrimer aza-bis-phosphonate (ABP) to modulate phenotype of monocytes (Mo) and monocytes derived dendritic cells (MoDC) activated in response to toll-like receptor 4 (TLR4) and interferon γ (IFN- γ) stimulation.

**Methods:**

Mo (*n* = 12) and MoDC (*n* = 11) from peripheral blood of healthy donors were prepared. Cells were preincubated or not for 1 hour with dendrimer ABP, then incubated with lipopolysaccharide (LPS; as a TLR4 ligand) and (IFN-γ) for 38 hours. Secretion of tumor necrosis factor α (TNFα), interleukin (IL) -1, IL-6, IL-12, IL-10 and IL-23 in the culture medium was measured by enzyme-linked immunosorbent assay (ELISA) and Cytokine Bead Array. Differentiation and subsequent maturation of MoDC from nine donors in the presence of LPS were analyzed by flow cytometry using CD80, CD86, CD83 and CD1a surface expression as markers.

**Results:**

Mo and MoDC were orientated to a pro-inflammatory state. In activated Mo, TNFα, IL-1β and IL-23 levels were significantly lower after prior incubation with dendrimer ABP. In activated MoDC, dendrimer ABP promoted IL-10 secretion while decreasing dramatically the level of IL-12. TNFα and IL-6 secretion were significantly lower in the presence of dendrimer ABP. LPS driven maturation of MoDC was impaired by dendrimer ABP treatment, as attested by the significantly lower expression of CD80 and CD86.

**Conclusion:**

Our data indicate that dendrimer ABP possesses immunomodulatory properties on human Mo and MoDC, in TLR4 + IFN-γ stimulation model, by inducing M2 alternative activation of Mo and promoting tolerogenic MoDC.

## Introduction

Cells from the myelo-monocytic lineage are involved in the pathophysiology of rheumatoid arthritis (RA) [1]. Macrophages and dendritic cells are thought to present arthritogenic peptides during the initial phase of the disease, leading to the activation of CD4+ T lymphocytes [2]. Macrophages also secrete pro-inflammatory cytokines (TNFα, IL-1 and IL-6), which are responsible for the inflammation symptoms. Macrophage-like synoviocytes (MLS) are derived from the monocytic lineage and participate in the production of cytokines and chemokines *in situ,* thus contributing to the proteolytic degradation of the cartilage [3]. In addition, osteoclasts are derived from a monocytic precursor [4].

Polarization of monocytes is a current concept derived from that of T cell polarization. Classically, the prototypical pro-inflammatory M1 polarization is obtained by activation mediated by LPS + IFN-γ or TNFα. Conversely, M2 polarization corresponds to a series of phenotypes obtained after a so-called “alternative” activation by IL-4 or IL-13 [5,6]. Although M1 produces IL-12, the different M2 phenotypes share high levels of IL-10 production. On a functional point of view, M2 macrophages are involved in immunoregulation and tissue reparation. Considering the characteristics of M1 and M2 phenotypes with regard to cytokine production, the IL-12:IL-10 balance has been proposed to evaluate the polarization of macrophages in inflammatory conditions [5].

Although their precise role in RA is not completely understood, dendritic cells (DCs) are most important in the initiation of the immune response. They are professional antigen presenting cells and also secrete pro-inflammatory cytokines, depending on the stimulation [7]. Besides their role in inflammation, tolerogenic DCs participate in immunoregulation through the production of indoleamine 2,3-dioxygenase (IDO), the production of IL-10, and the regulated expression of their co-stimulatory molecules, such as CD80 and CD86 [8]. Their role in RA could thus be different according to the pattern of stimulation in their environment.

Toll-like receptors (TLR) expressed at the surface of Mo and DC are responsible for early, powerful and non-specific immune reaction in response to danger signals. They are capable of initiating adaptive immune response as well as regulating co-stimulation, cytokine and chemokine secretion [9]. A central role of TLR in the initiation and/or maintenance of RA has been proposed. On the one hand, TLR2 and 4 are capable of recognizing endogenous proteins among which some are of synovial origin (Hsp60, fibrinogen, hyaluronic acid) [10-16]. On the other hand, TLR2 and 4 recognize infectious targets either latent or persistent in the organism [17-22]. TLRs have the capacity to initiate and maintain an inflammatory reaction in response to endogenous danger signals and thus represent good candidates for the long-term inflammation of RA [23]. The induction of co-stimulation molecules on APC by TLR may contribute to the initiation phase of the disease as well as to the vicious circle of inflammation following the as yet unidentified triggering phenomenon [23].

**Figure 1:**
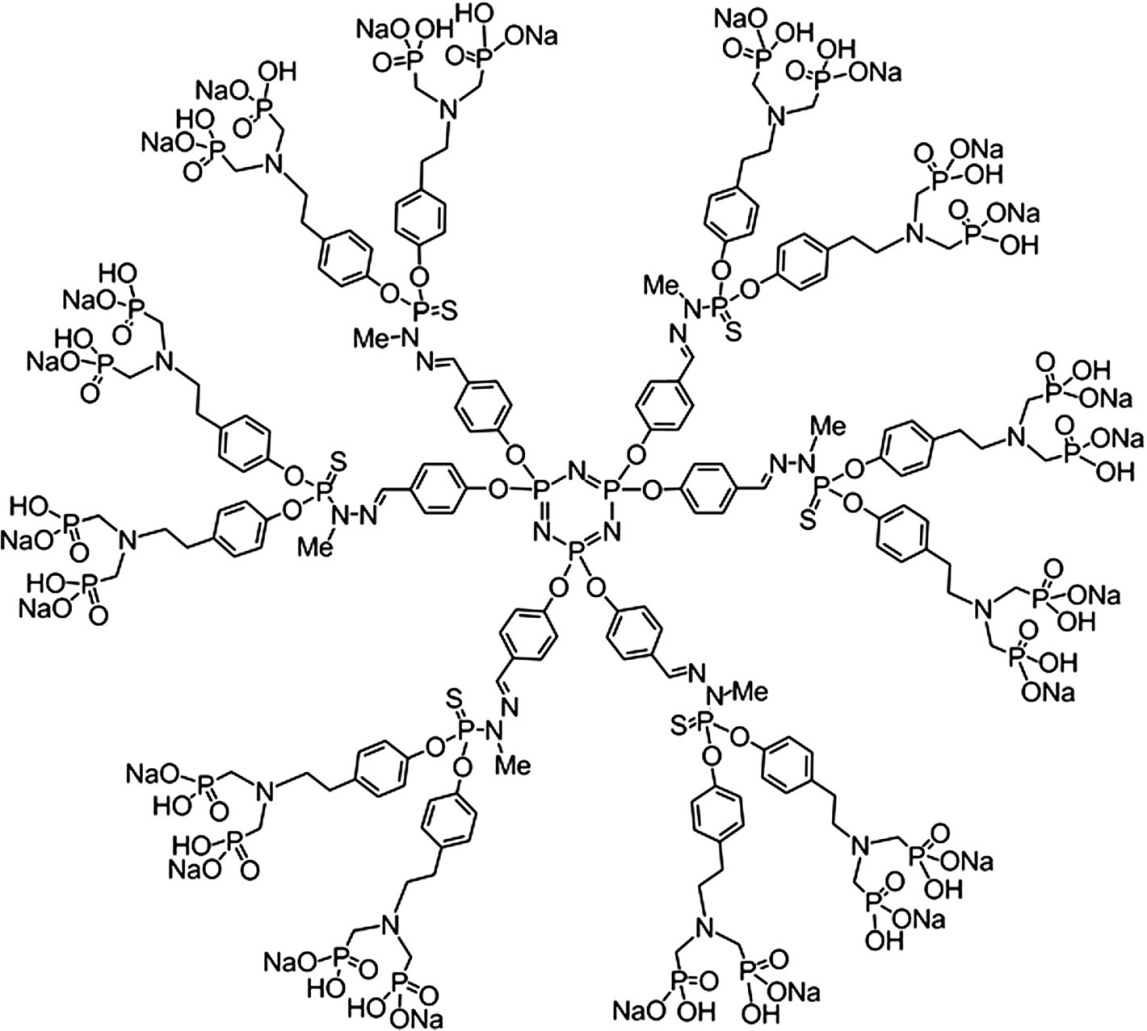
Structure of dendrimer azabisphosphonate (mw = 5,820 da).

Dendrimer ABP (aza-bis-phosphonate) is a synthetic hyper-branched nanomolecule which belongs to the family of phosphorus-containing dendrimers (Figure 1) [24]. Dendrimer ABP has shown promising results in the treatment of experimental arthritis [25]. We have demonstrated in two murine models that this dendrimer inhibits arthritis and bone erosion by decreasing pro-inflammatory cytokines, increasing anti-inflammatorycytokines as well as inhibiting osteoclastogenesis [25]. Moreover, in human cultures, dendrimer ABP has shown immunomodulatory properties targeted to Mo leading to M2 activation [26], amplification of IL-10 producing CD4 T cells [26]. However, this has not been previously tested in pro-inflammatory conditions.

IFN-γ has been shown to prime cells for TLR4 induction of pro-inflammatory cytokines [27]. In this current study, we have analyzed the potential mechanism through which dendrimer ABP may act to modulate inflammation. We thus have chosen to orientate Mo as well as DC towards an anti-inflammatory state using dendrimer ABP before applying a classical pro-inflammatory protocol using TLR4 ligand LPS in the presence of IFN-γ. Pro-inflammatory cytokines supposed to be involved in RA, such as TNFα, IL-1β, IL-6, IL-12 and IL-23, were studied. Anti-inflammatory cytokine IL-10 involved in regulation of the immune response and in tolerogenic phenotype of DC was also examined.

## Materials and methods

### Isolation of monocytes

Peripheral blood mononucleated cells (PBMCs) were obtained from normal blood donors of the Etablissement Français du Sang (EFS), Toulouse, France. Informed written consent was obtained from all blood donors, and the study protocol was approved by the INSERM-EFS ethical committee. Briefly, CD14+ monocytes were purified from PBMC isolated on Pancoll (Pan Biotech, Aidenbach, Germany) by negative sorting using Dynabeads (Dynabeads Untouched Human Monocytes, Invitrogen, Oslo, Norway). Purity was measured by flow cytometry (FC-500 Beckman Coulter, Brea, CA, USA) using a CD14-FITC antibody (clone HCD14, Bio Legend, San Diego, CA, USA). Data were analyzed using FlowJo (Tree Star). Purity was >90%.

### Culture and activation of monocytes

Monocytes were cultured at 37°C, 5% CO_2_, 100% humidity in RPMI-1640-Glutamax (Gibco; Paisley, UK) containing 10% fetal calf serum (FCS) (Gibco), penicillin (100 U/ml; Gibco), streptomycin (100 μg/ml; Gibco), non-essential amino acids (1%; PAA, Pasching, Austria), and sodium pyruvate (1%; Sigma-Aldrich).

Monocytes were first incubated with dendrimer ABP (10 μM) for one hour. They were then activated in the presence of LPS from *E. coli* (20 ng/ml; Sigma-Aldrich) and IFN-γ (250 U/ml) [28] for 38 hours for optimal cytokine production. No cell death was observed in the presence of dendrimer ABP, as expected from the study by Poupot *et al*. [24], using flow cytometry Forward Scatter and Side Scatter.

### Generation and activation of monocyte-derived dendritic cells (MoDC)

Immature MoDC were generated according to Sallusto and Lanzavecchia [29]. Briefly, monocytes (0.8 × 10^6^/ml) were cultured in the presence of rIL-4 (50 ng/ml; PeproTech, Rocky Hill, NJ, USA) and rGM-CSF (100 ng/ml; PeproTech) for six days. Culture media was complete 10% FCS RPMI-1640. The phenotype was analyzed on FC-500 Coulter flow cytometer using the following antibodies anti CD14-FITC (clone HCD14, BD Biosciences), CD11c-APC (clone 3.9, Bio Legend), CD1a-APC (clone HI149, BD Biosciences), CD80-PE (clone 2D10, Bio Legend), CD83-PerCP Cy5.5 (clone HB15e, Bio Legend), CD86-Alexa Fluor 488 (clone IT2.2, Bio Legend) and corresponding isotype controls. Data were analyzed using FlowJo (Tree Star).

MoDC were cultured at the final concentration of 0.2 × 10^6^/ml in complete 10% FCS RPMI-1640 media and treated as Mo with regard to activation.

### Production of cytokines

Cultured supernatants were collected and kept at -80°C until analysis. Concentrations of IL-1β, IL-6, IL-10, IL-12 and TNFα were determined simultaneously using Cytometric Bead Array (Human Inflammatory Cytokine Kit, BD Biosciences). Data acquisition was performed on a FACSCalibur (BD Biosciences) and analysis was performed using FCAP Array v3 (Soft Flow). IL-23 was quantitated by ELISA (Ready-SET-Go, eBioscience, San Diego, CA, USA) on a Varioskan Flash (Thermo Scientific) spectrophotometer and analyzed using the SkanIt™ (Thermo Scientific) program.

### Statistical analysis

Statistical analyses were performed using Stata 12 (Statacorp LP). A bilateral paired Student’s *t* test was performed when variables followed a Gaussian distribution (Shapiro-Wilk test). A paired rank Wilcoxon test was used when applicable. Statistically, *P* <0.05 was considered as significant with 95% interval confidence.

**Figure 2:**
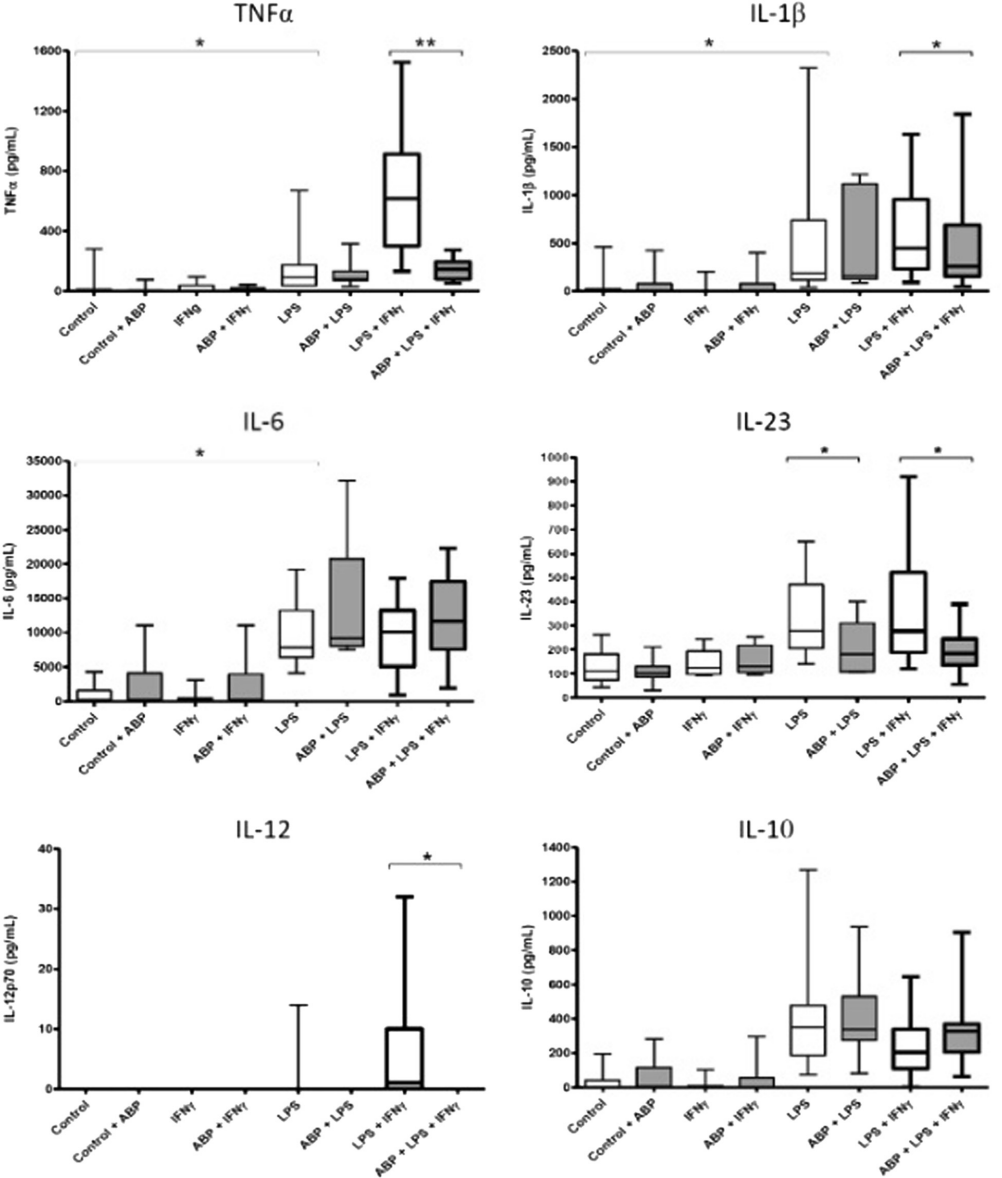
**Actions of dendrimer ABP on cytokine production by monocytes.** TNFα, IL-1β, IL-6, IL-23, IL-12 and IL-10 were measured in the culture supernatant of activated Mo from 12 donors. Data are presented as box plots. Activation was performed, as indicated, in the presence or in the absence of dendrimer ABP. *P < 0.05; **P = 0.0005. ABP, aza-bis-phosphonate; IL, interleukin; Mo, monocytes; TNF, tumor necrosis factor.

**Figure 3:**
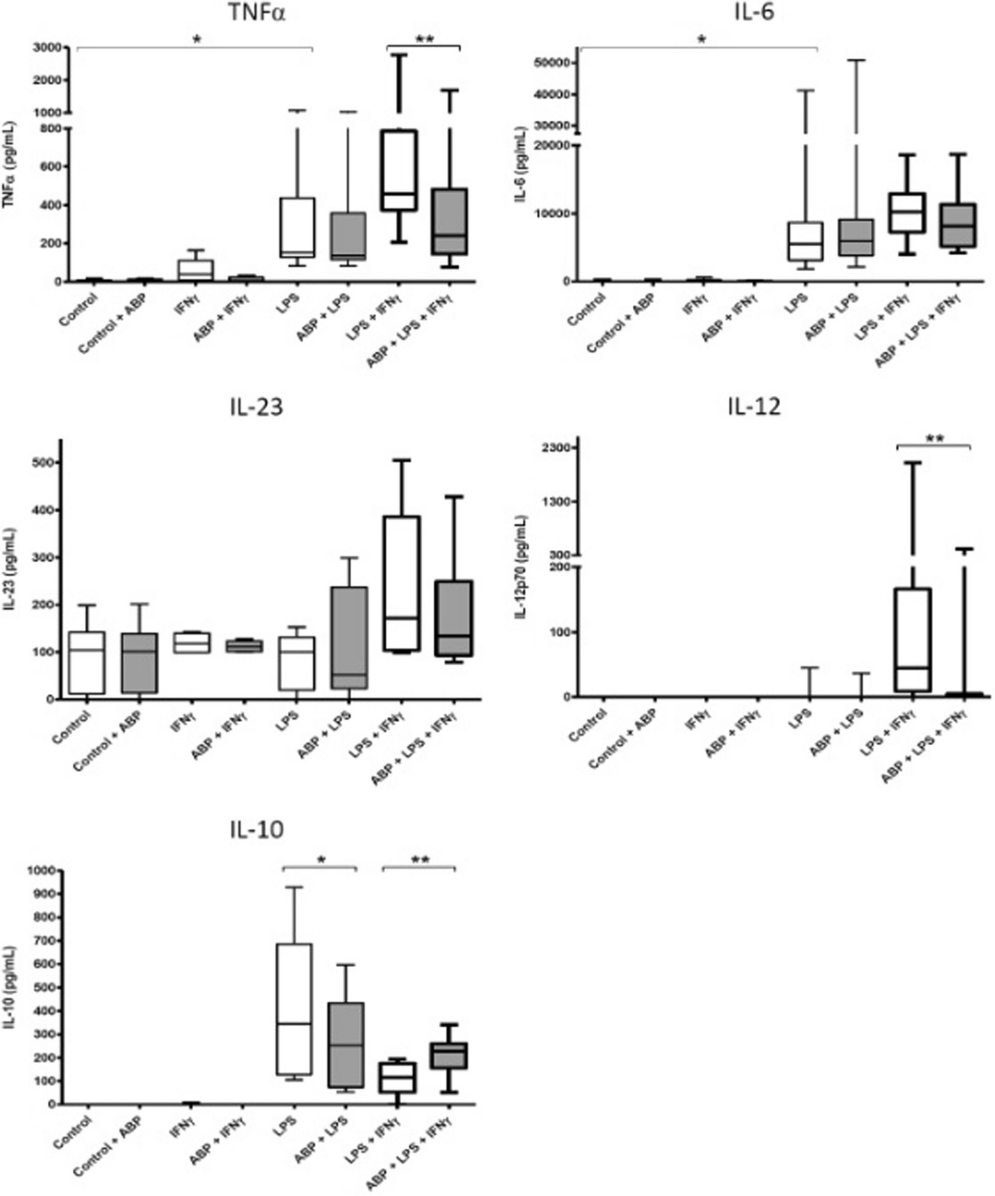
**Actions of dendrimer ABP on cytokine production by MoDC.** TNFα, IL-6, IL-23, IL-12 and IL-10 were measured in the culture supernatant of activated MoDC from 11 donors. Data are presented as box plots. Activation was performed, as indicated, in the presence or in the absence of dendrimer ABP. *P <0.05; **P <0.005. ABP, aza-bis-phosphonate; IL, interleukin; MoDC, monocyte derived dendritic cells; TNF, tumor necrosis factor.

Box plots (Figures 2 and 3) represent the 25^th^ and 75^th^ percentiles (interquartile range, IQR). The ends of the whiskers are the lowest and highest values, respectively.

**Figure 4:**
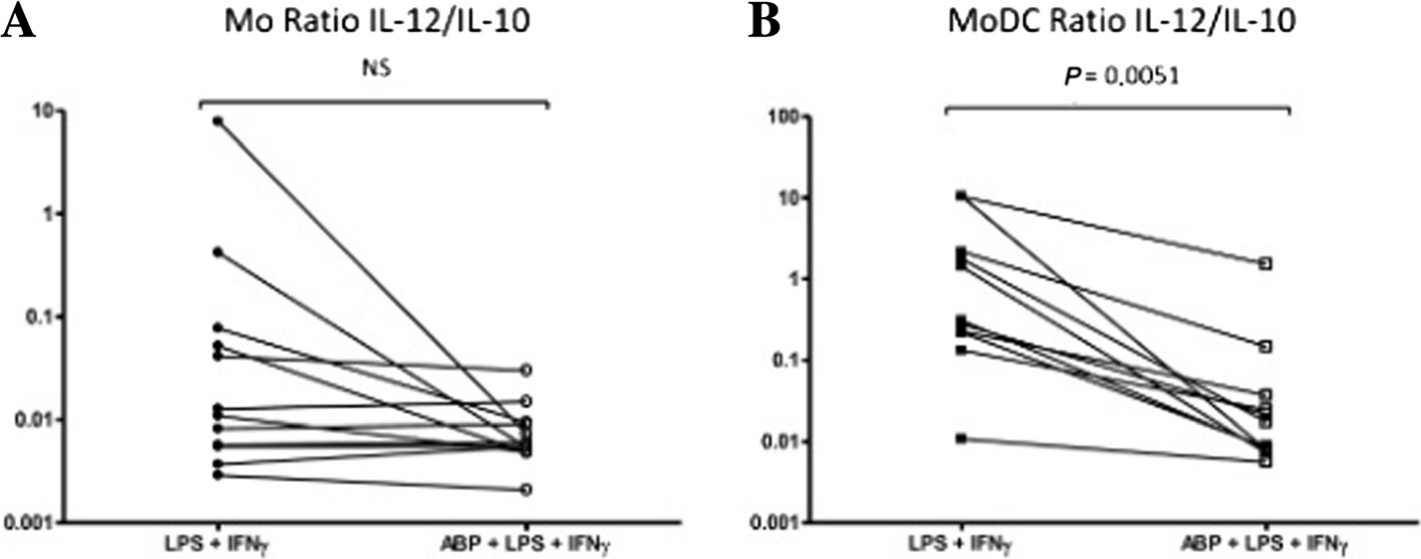
**Dendrimer ABP favors the IL-10 over IL-12 production in MoDC.** The ratio of IL-12 to IL-10 production in the presence or in the absence of ABP and LPS + IFN-γ +/− dendrimer ABP was calculated for Mo **(A)** and MoDC **(B)**. When IL-10 levels were not detectable, detection limit (5 pg/mL) was chosen as the denominator of the ratio. ABP, aza-bis-phosphonate; IFN-γ, interferon-gamma; IL, interleukin; LPS, lipopolysaccharides; Mo, monocytes; MoDC, monocyte derived dendritic cells.

When data did not follow normal distribution (Figure 4C) SD or SEM could not be calculated. Bars represent the 75^th^ percentile.

## Results

### Actions of dendrimer ABP on pro-inflammatory cytokine production by activated monocytes

The structure of dendrimer ABP (mw = 5,820 Da) is shown in Figure 1[24-26]. We evaluated the secretion of TNFα, IL-1β, IL-6, IL-23 and IL-12 by monocytes from 12 healthy blood donors in 12 independent experiments. Monocytes were pre-incubated in the presence or the absence of dendrimer ABP, and then activated by IFN-γ, LPS and the association of LPS + IFN-γ, which is known to induce a typical M1 phenotype [5]. Data are presented as box plots in Figure 2 and in Table 1.

**Table 1:** Summary of cytokine secretion by Mo and MoDC

**Cytokines (pg/ml)**	**Control; median (IQR)**	**LPS + IFN-γ; median (IQR)**	**LPS + IFN-γ + ABP; median (IQR)**	***P***
**Mo IL-12**	0 (0; 0)	1 (0; 10)	0 (0; 0)	0.0159
**Mo IL-10**	0 (0; 5)	205 (110; 340)	328 (206; 369)	ns
**Mo TNFα**	0 (0; 6)	617 (302; 912)	147 (82: 195)	0.0005
**Mo IL-1β**	0 (0; 4)	448 (232; 953)	261 (155; 688)	0.0186
**Mo IL-6**	175 (57; 403)	10,059 (5,074; 13,251)	11,682 (7,537; 17,425)	ns
**Mo IL-23**	115 (99; 181)	277 (188; 521)	184 (137; 244)	0.036
**MoDC IL-12**	0 (0; 0)	45 (9; 166)	0 (0; 5)	0.0038
**MoDC IL-10**	0 (0; 0)	117 (53; 176)	228 (157; 260)	0.001
**MoDC TNFα**	0 (0; 5)	458 (374; 786)	241 (145, 481)	0.0033
**MoDC IL-1β**	0 (0; 0)	0 (0; 0)	0 (0, 8)	ns
**MoDC IL-6**	13 (0; 29)	10,255 (7,262, 12,864)	8,131 (5,160; 11,310)	ns
**MoDC IL-23**	130 (41; 152)	172 (104; 386)	134 (93; 249)	ns

Cytokines were measured in the supernatant of Mo and MoDC cultures. Concentrations were measured in the absence of stimulation (control) and after activation with LPS + IFN-γ with or without prior incubation with dendrimer ABP. Results are from 12 (Mo) and 11 (MoDC) blood donors, respectively. ABP, aza-bis-phosphonate; IFN-γ, interferon-gamma; IL, interleukin; IQR, interquartile range; LPS, lipopolysaccharide; Mo, monocytes; MoDC, monocyte derived dendritic cells.

IFN-γ alone was not able to induce any studied cytokine. Dendrimer ABP alone induced only a modest and non-statistically significant production of IL-6. LPS alone was sufficient to induce significant secretion of TNFα , IL-1β and IL-6 by monocytes, but the association LPS + IFN-γ was required to obtain higher production of TNFα. IL-12 secretion was obtained only with the combination of LPS + IFNγ, as expected from references [30,31], with a very heterogeneous response among the different samples.

Dendrimer ABP at 10 μM induced a profound inhibition of the production of TNFα (*P* = 0.0005) but also a significant decrease of the secretion of IL-1β (*P* = 0.0186). On the opposite side, we observed a slight but non-significant increase of IL-6 under the action of dendrimer ABP after stimulation of monocytes by LPS and by LPS + IFN-γ. IL-12 production was completely inhibited by dendrimer ABP (*P* = 0.0159).

IL-23 production is mostly due to monocytes and DC activated by danger signals, such as those provided by TLRs [32]. Due to the importance of Th17 in arthritis, IL-23 production was investigated. Secretion of IL-23 by monocytes was induced in eight independent experiments (Figure 2 and Table 1). Stimulation with LPS and with LPS + IFN-γ induced IL-23 secretion. In both cases, pre-incubation with dendrimer ABP induced a significantly lower production (respectively, *P* = 0.0431 and *P* = 0.0360). Stimulation by IFN-γ alone did not induce IL-23 production above control. Dendrimer alone did not induce IL-23 secretion.

### Actions of dendrimer ABP on pro-inflammatory cytokine production by MoDC

Cytokine production by activated MoDC in 11 donors was measured in the same conditions as with monocytes (Figure 3) but IL-1β production was never observed, whatever the conditions (Table 1). LPS alone induced significant production of TNFα and IL-6. Dendrimer alone was never able to induce the secretion of the different cytokines. Again, dendrimer ABP has a clear-cut effect on TNFα, decreasing the production induced by LPS + IFN-γ (*P* = 0.0033).

Only the LPS + IFN-γ stimulation was able to induce IL-23 production. However, this production was not significantly diminished by dendrimer ABP. As we observed with monocytes, MoDC produced IL-12 after stimulation with LPS and IFN-γ, with a heterogeneous response, and this production was totally inhibited by dendrimer ABP.

Stimulation by LPS or LPS + IFN-γ induced a significant IL-6 production. However, no significant decrease of IL-6 was observed in the presence of dendrimer ABP.

### Dendrimer ABP favors IL-10 over IL-12 production in MoDC

**Figure 5:**
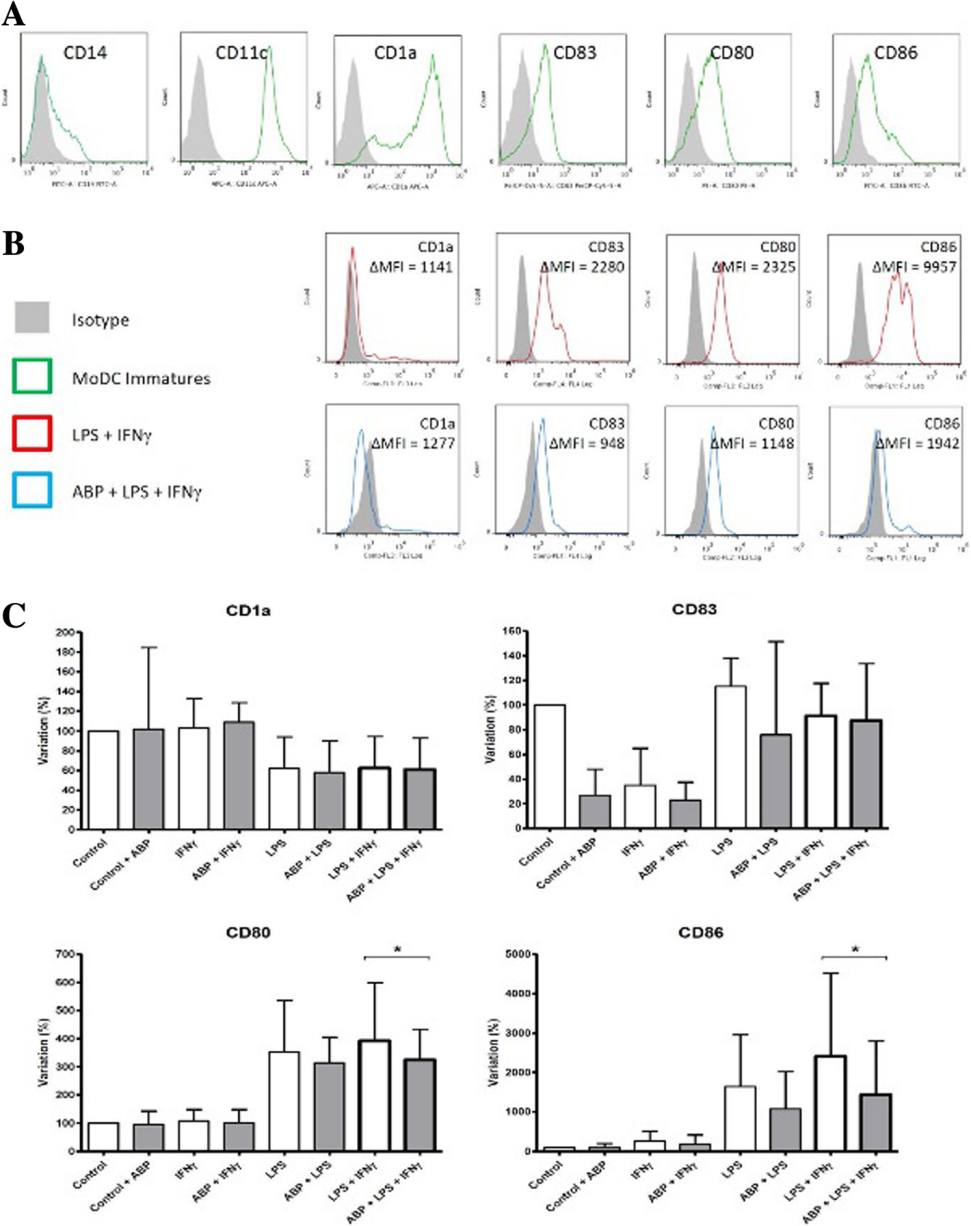
**Modulation of MoDC maturation by dendrimer ABP. A**. the phenotype of immature MoDC was determined by flow cytometry. Expression of CD14, CD1a, CD11c, CD83, CD80 and CD86 was evaluated after six days of differentiation in the presence of IL-4 and granulocyte-macrophage colony-stimulating factor (GM-CSF). These results are representative of nine donors. **B**. Phenotype of mature MoDC was determined by flow cytometry. Markers of MoDC were evaluated after incubation for 38 hours in the presence or in the absence of dendrimer ABP and LPS + IFN-γ. The phenotype of non-manipulated, immature MoDC is shown as the control. These results are representative of nine donors. Mean Fluorescence Intensity (MFI) differences between staining and isotype control are shown. **C**. Variation of expression of MoDC maturation markers CD1a, CD83, CD80 and CD86. MFI was used to measure the level of expression of markers. Data are presented as the relative percentage of expression, as compared to controls obtained in the absence of stimulation (100%). Bars correspond to the 75^th^ percentile (IQR). Data are from nine donors. *P <0.05. ABP, aza-bis-phosphonate; IFN-γ, interferon-gamma; IQR, interquartile range; LPS, lipopolysaccharides; MoDC, monocyte derived dendritic cells.

In DC, the production of IL-10 is characteristic of tolerogenic DC which possess an immunomodulatory anti-inflammatory activity. We have analyzed the secretion of both cytokines in MoDC from 11 blood donors, in 11 independent experiments (Figure 5 and Table 1). In accordance with the literature, the level of IL-10 production by MoDC was weakest in the presence of IFN-γ, which is known to inhibit IL-10 [33]. The effect of dendrimer was different according to the mode of cell activation. In the absence of IFN-γ (LPS alone) the production of IL-10 by MoDC was significantly diminished (*P* = 0.0135). However, in the LPS + IFN-γ stimulation, IL-10 production was down-regulated by IFN-γ compared to LPS alone. Dendrimer ABP prevented down-regulation of IL-10 induced in the presence of IFN-γ during TLR4 stimulation (*P* = 0.001).

Similar to Mo, IL-12 production was obtained only in the presence of LPS + IFNγ; the level of IL-12 was significantly lower in the presence of dendrimer ABP.

The resulting IL-12:IL-10 ratio was significantly decreased in MoDC stimulated by LPS + IFN-γ and pretreated by dendrimer ABP (*P* = 0.0051) (Figure 4B). This is strongly in favor of an anti-inflammatory activity.In monocytes, a decrease of the IL-12:IL-10 ratio in the presence of dendrimer was observed but did not reach statistical significance although donors with the highest initial IL-12:IL-10 ratios were the ones which were the most affected by dendrimer ABP (Figure 4A).

### Dendrimer ABP affects the phenotype of TLR4-activated MoDC

In addition to the TCR stimulation, two other signals are indispensable for the activation of T lymphocytes: engagement of co-stimulation molecules and cytokine signaling. We thus have looked at the expression of CD80 and CD86 by MoDC in flow cytometry experiments. A decrease of CD1a and an increase of CD83 were also examined as markers of maturation. Phenotype of MoDC was controlled and found to be CD14-, CD11c+, CD1a+, CD80low and CD86low (Figure 5A). The maturation phenotype of MoDC in the presence of LPS + IFN-γ was assessed. Figure 5 shows the classical maturation phenotype obtained from nine donors in nine independent experiments (decrease of CD1a, induction of CD83, CD80, CD86). CD80 and CD86 expression was significantly lower in the presence of dendrimer ABP (*P* <0.05) (Figure 5B, C). The expression of CD83 and CD1a was not altered by dendrimer ABP. Thus, co-stimulation may be diminished in DC in the presence of the immunomodulatory agent dendrimer ABP.

## Discussion

In this current work, we have studied how ABP dendrimer can orientate Mo and MoDC toward phenotypes capable of fighting classical pro-inflammatory stimulus mediated by LPS and IFN-γ. We showed that dendrimer ABP displays an anti-inflammatory activity by favoring the M2 phenotype and by preventing TNFα and IL-23 production by Mo in the context of pro-inflammatory stimulus. In MoDC, dendrimer ABP induced a tolerogenic phenotype by favoring the secretion of IL-10 and decreasing that of IL-12 and TNFα. Reduced expression of CD80 and CD86 was also observed. Our data suggest an immunomodulatory role of dendrimer ABP on both Mo and MoDC. This may be of importance in breaking the vicious circle of inflammation in the treatment of arthritis.

Activated monocytes/macrophages are the main producers of TNFα and IL-1β in the rheumatoid synovium. The decrease of TNFα production by Mo and MoDC is of importance with regard to RA treatment. On the one hand, TNFα is a key mediator of synovial inflammation and bone erosion [4,34]. On the other hand, it induces the production of IL-1β, which is also responsible of bone loss [35]. Whatever the mechanism, by a direct effect on IL-1β production or an indirect action through the inhibition of TNFα, dendrimer ABP induced a decrease of IL-1β production in Mo.

In Mo and MoDC put in the presence of LPS + IFN-γ, dendrimer ABP also decreased the production of IL-12 and increased IL-10, although the IL-12:IL-10 ratio was significantly decreased only in MoDC. The decrease of the IL-12:IL-10 ratio may also be in part responsible for the decrease of CD80 and CD86 expression in MoDC. In Mo, IL-12:IL-10 ratios were much lower than in MoDC and may have been difficult to modulate. Indeed, only blood donors with the highest IL-12:IL-10 ratio in Mo showed a decrease after ABP treatment. Thus, an alternative activation of Mo and MoDC is induced by dendrimer ABP. This is in accordance with previous publications on mRNA expression in Mo [26].

IL-10 is an anti-inflammatory cytokine which induces, among others, regulatory T cells Tr1 [36] and, in DC, the decrease of co-stimulatory molecules, the inhibition of maturation, of antigen presentation and of pro-inflammatory cytokines production [33]. There is a competition between intracellular signaling of IL-10 and IFN-γ. IL-10 has been shown to inhibit STAT1 signaling and the transcription of genes of the “IFN-γ signature”. On the opposite side, IFN-γ inhibits the ERK- and p38-dependent production of IL-10 by blocking the STAT3 signaling [27,37]. IL-10 production by MoDC was increased by dendrimer ABP in the context of LPS + IFN-γ signaling. We may thus hypothesize that dendrimer ABP decreases the inhibitory action of IFN-γ on IL-10 production. This is particularly illustrated by the fact that, in MoDC, ABP alone (or in the presence of LPS) did not induce significant production of IL-10 in the absence of IFN-γ. If the orientation of dendrimer ABP-treated MoDC is tolerogenic, this should have a beneficial effect with regard to anti-RA therapeutic objectives. Thus, our current data are more informative with regard to MoDC, which are highly relevant cells in RA [38]. This trend towards IL-10 production has already been observed in arthritic mice treated with dendrimer ABP [25] and in a rat model of uveitis [39]. We make the hypothesis that dendrimer ABP possesses stronger immunomodulatory properties when IFN-γ stimulation is engaged. This is supported by the observation of dendrimer ABP effect on IL-1β in Mo and IL-6 in MoDC in the LPS + IFN-γ, but not in the context of LPS alone. The mechanism of action of dendrimer ABP on the JAK-STAT pathway remains to be investigated.

DC are most important in antigen presentation and orientation of the T cell immune response. Dendrimer ABP may participate in the immunomodulation as well, by acting on co-stimulation (decrease of CD80 and CD86) and decrease of cytokine secretion. Future experiments will determine whether the decrease of CD80 and CD86 and the decrease of IL-12 production have an impact on the T helper cell compartment.

The role of IFN-γ in RA is controversial. On the one hand, IFN-γ activates Mo/macrophages in the synovium [1]. On the other hand, mice deficient in IFN-γ receptors have accelerated collagen-induced arthritis [40] and IFN-γ inhibits the development of osteoclasts [4] and of Th17 cells [41], which are major players in arthritis. In this current study, we have used IFN-γ along with the TLR4 agonist LPS as classical tools to activate Mo to M1 polarization and induce cytokine production. Whether this reflects precisely the activation *in vivo* in RA is beyond the scope of our study.

Most of the current treatments for RA are aimed at neutralizing inflammatory cytokines. Blocking TNFα was shown in early publications to result in the inhibition of IL-1β, IL-6 and IL-8 production, thus putting TNFα at the center stage of inflammatory cytokine regulation and providing a rationale for the use of anti-TNF reagents. It is thus of interest that inhibition of TNFα production was a prominent feature of the effect of dendrimer ABP on both Mo and MoDC, although it was not followed by inhibition of IL-1β in MoDC and by inhibition of IL-6. It is of note that our setting allows for the analysis of isolated populations. However, in our previous study, the resulting effect of dendrimer ABP action in arthritic mice was a dramatic decrease of both IL-1β and IL-6 [25], suggesting a strong *in vivo* capability to decrease inflammatory cytokines involved in arthritis. In addition, our current work shows that IL-12 production was decreased in Mo and MoDC. However, IL-23 was significantly decreased only in Mo.

## Conclusion

We have added to the understanding of mechanisms of action of a potential therapeutic agent dendrimer ABP. We have confirmed that dendrimer ABP orientates Mo towards an M2 phenotype and shown that orientation of MoDC is towards a tolerogenic phenotype. Our data are in accordance with previous observations from our laboratory showing immunomodulatory and anti-osteoclastic effects [25,26]. Our data add further arguments for dendrimer ABP as a potential therapeutic agent ofor RA.

## Abbreviations

ABP: Aza-bis-phosphonate; DC: Dendritic cells; FCS: Fetal calf serum; IFN-γ: Interferon gamma; IL: Interleukin; IQR: Interquartile range; LPS: Lipopolysaccharide; MFI: Mean Fluorescence Intensity; Mo: Monocytes; MoDC: Monocytes-derived dendritic cells; PBMCs: Peripheral blood mononucleated cells; RA: Rheumatoid arthritis; TLR4: Toll-like receptor 4; TNFα: Tumor necrosis factor alpha.

## Competing interests

The authors declare that they have no competing interests.

## Authors’ contributions

YD contributed to the study conception and design, data collection and analysis, manuscript writing and final approval of the manuscript. SF, MB and DN were responsible for data collection and analysis, critical revision and final approval of the manuscript. COT and AMC contributed to conception and design, critical revision and final approval of manuscript. RP, AC and JLD contributed to conception and design, data analysis, manuscript writing and final approval of the manuscript. All authors read and approved the final manuscript. All authors have given final approval of the version to be published. They agree to be accountable for all aspects of the work in ensuring that questions related to the accuracy or integrity of any part of the work are appropriately investigated and resolved.
